# Using a consensus meeting to enhance fracture care education in low income countries

**DOI:** 10.1186/s12909-023-04077-8

**Published:** 2023-02-15

**Authors:** Zahra Jaffry, Maureen Sabawo, William J. Harrison, Alexander T. Schade

**Affiliations:** 1 Bart’s Health NHS Trust, London, UK; 2 Kamuzu University of Health Sciences, Blantyre, Malawi; 3 Countess of Chester Hospital NHS Trust, London, UK; 4 AO Alliance Africa, Davos, Switzerland; 5 Malawi- Liverpool Wellcome Trust, Blantyre, Malawi; 6 Liverpool School of Tropical Medicine, Liverpool, UK

**Keywords:** Fracture management, Clinical education, Consensus meeting, Nominal group technique, Low middle income countries

## Abstract

**Background:**

A key strategy to building surgical capacity in low income countries involves training care providers, particularly in the interventions highlighted by the Lancet Commission for Global Surgery, including the management of open fractures. This is a common injury, especially in areas with a high incidence of road traffic incidents. The aim of this study was to use a nominal group consensus method to design a course on open fracture management for clinical officers in Malawi.

**Methods:**

The nominal group meeting was held over two days, including clinical officers and surgeons from Malawi and the UK with various levels of expertise in the fields of global surgery, orthopaedics and education. The group was posed with questions on course content, delivery and evaluation. Each participant was encouraged to suggest an answer and the advantages and disadvantages of each suggestion were discussed before voting through an anonymous online platform. Voting included use of a Likert scale or ranking available options. Ethical approval for this process was obtained from the College of Medicine Research and Ethics Committee Malawi and the Liverpool School of Tropical Medicine.

**Results:**

All suggested course topics received an average score of greater than 8 out of 10 on a Likert scale and were included in the final programme. Videos was the highest ranking option as a method for delivering pre-course material. The highest ranking methods for each course topic included lectures, videos and practicals. When asked what practical skill should be tested at the end of the course, the highest ranking option was “initial assessment”.

**Conclusion:**

This work outlines how a consensus meeting can be used to design an educational intervention to improve patient care and outcomes. Through combining the perspectives of both the trainer and trainee, the course aligns both agendas so that it is relevant and sustainable.

## Background

The path to improving surgical capacity lies in addressing the inequity in the distribution of resources. This is evident in that Africa and southeast Asia have only 12% of the global specialist surgical workforce and only 6% of surgeries worldwide take place here, despite holding a third of the world’s population [1, 2]. In Malawi, specifically, this equates to 0.019 physicians per 1,000 people compared to the World Health Organisation (WHO) standard of 2.5 physicians per 1,000 [3]. The proposed solution for this involves the use of other health care professionals that have been trained to perform the tasks of doctors and surgeons, such as clinical officers, who have been shown to perform just as well after spending a shorter amount of time in training [4, 5]. Supporting these groups is an important strategy, particularly through training in the interventions highlighted by organisations such as the Lancet Commission for Global Surgery, dedicated to improving access to surgery. This includes the management of open fractures (where there is a break in the skin at the site of the broken bone) [1].

Open fractures, associated with complications such as infection (18%), non-union (15%), amputation (15%), overall poor function and catastrophic loss of income, are a common consequence of road traffic incidents and disproportionately affect low and middle income countries (LMICs) [6]. Malawi, specifically, has the ninth highest rate of road traffic deaths in the world at 31/100,000 [7], with 16% of all fractures being caused by this mechanism of injury [8]. Of these fractures, 12% are open [8]. The Malawi Orthopaedic Association (MOA) and AO Alliance (an organisation strengthening the care of the injured in LMICs), have created open fracture guidelines to improve patient outcomes [9].

A plan to support effective implementation of these guidelines involves creating a course that is pragmatic for those treating injuries in a low resource setting while including the insights and agendas of both trainers (surgeons and senior clinical officers) and trainees (clinical officers). A way in which to do this is through a consensus that would allow shared decision making on course content, delivery and evaluation [10].

There are a number of consensus development methods. The most widely recognised include the Delphi, conference and nominal group techniques [10, 11]. The first involves the use of mailed questionnaires, the responses to which are summarised and sent back to participants with another questionnaire. This process can be repeated till a level of agreement is reached [10]. The second involves a chaired open group discussion following a presentation of related evidence by experts [10]. The third involves a structured facilitated group meeting where individuals present options to discuss. Participants then vote for their preference [10, 11].

The aim of this study was to use a nominal group consensus method to design a course on open fracture management for clinical officers in Malawi. It describes how the meeting was conducted and its outcomes as a guide for future projects, outlining the logistical considerations involved and the lessons learnt.

## Methods

The group consisted of a total of 16 people, including four surgeons and nine clinical officers representing different districts in Malawi and three surgeons from the UK, all with various levels of expertise in the fields of global surgery, orthopaedics and education. More specifically, two participants had a masters and one had a post graduate certificate in clinical education. Three were trainers and course developers for the diploma in orthopaedics for clinical officers in Malawi and another three were faculty trainers and mentors for the AO Alliance. The group was invited to the two day meeting in a venue in Malawi. Two of the surgeons from the UK joined the meeting virtually.

**Fig. 1:**
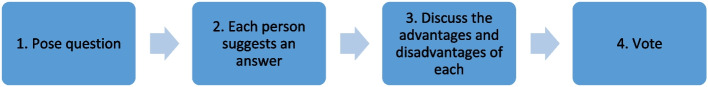
Steps involved in running the nominal group consensus meeting

The room had been set up in a u-shape to allow clear views of participants as well as the projection screen and a flipchart at the front. The first day began with an introduction to the MOA/AO Alliance open fracture guidelines and preliminary findings from an audit on compliance with them and patient outcomes. The purpose of the meeting was explained, specifically a need for input from both trainers and trainees to design a course that would allow clinical officers to understand and implement these guidelines effectively in their clinical practice. The way in which the meeting would run was also explained (Fig. 1).

The following four questions were posed over the course of the two days in turn: 1. What topics should be covered? 2. What method should be used to deliver pre-course material (an overview of the topics covered in the course)? 3. What methods should be used to deliver course material? 4. What practical skill should be assessed? After each participant had made a suggestion to a question, the advantages and disadvantages of each were discussed and recorded on the flip chart. Course conveners who previously had the opportunity to research various other methods also made suggestions that had not been mentioned already to discuss in the same way. The suggestions were added to a questionnaire on Microsoft Forms [12] that could be accessed via both a link and QR code. The response screen was shared at the front of the room to ensure all had voted before proceeding.

Voting for each question was conducted through either the use of a Likert scale (1–10) or ranking the options in order of preference. The meeting ended on day two after a presentation of the final results and a preliminary course outline on which participants then had a final opportunity to provide feedback.

This study has been approved by the College of Medicine Research and Ethics Committee Malawi (P.09/20/3130) and the Liverpool School of Tropical Medicine (20–068).

## Results

The suggestions made by the group as well as the results of anonymous voting for each question are presented below.

### Question 1. What topics should be covered?

**Fig. 2:**
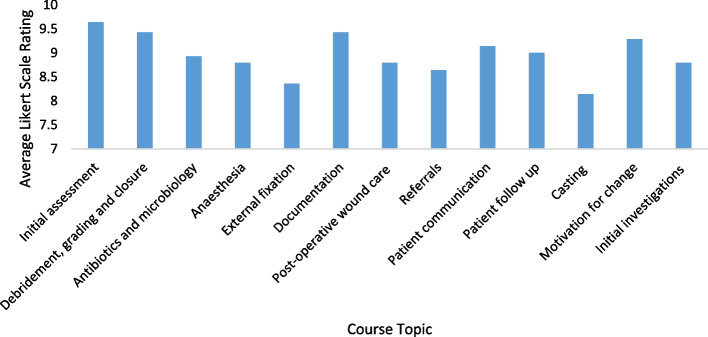
The average Likert scale rating for each suggested topic in answer to question 1, where 10 represents the highest level of agreement that the topic should be included in the course

The following topics were suggested for inclusion on the course: 1. Initial assessment, 2. Debridement, grading and closure, 3. Antibiotics and microbiology, 4. Anaesthesia, 5. External fixation, 5. Documentation, 5. Post-operative wound care, 6. Referrals, 7. Patient communication, 8. Patient follow ups, 9. Casting, 10. Motivation for change, 11. Initial investigations. The average Likert scale rating for all topics was greater than 5, where 10 represents the highest level of agreement (Fig. 2), therefore all were included in the final course programme.

### Question 2. What method should be used to deliver pre-course material (an overview of the topics covered in the course)?

Six pre-course delivery methods were suggested. Participants ranked options in order of highest to lowest preference. Each option was given a score from 1–6 depending on how it was ranked by each participant. Scores were tallied to determine the final rank order from highest to lowest preference amongst the group: 1. Video, 2. Infographic, 3. PDF (Portable Document Format), 4. Pre-test, 5. E-learning, 6. Booklet.

### Question 3. What methods should be used to deliver course material?

A number of delivery methods were suggested for each course topic and so options were ranked in order of preference from highest to lowest as outlined in Table 1. The final order was determined as it was for question 2.

**Table 1:** Chosen delivery methods for each course topic in order of preference from highest to lowest

Course Topic	Delivery Options (highest to lowest preference)
Initial assessment	lecture
	video
	practical
	group session
Debridement, grading and closure	video
	lecture
	practical
Antibiotics and microbiology	lecture
	booklet
	video
Anaesthesia	lecture
	video
	practical
External fixation	practical
	lecture
	video
Documentation	lecture
	case based discussion
Post-operative wound care	lecture
	booklet/infographic
Referrals	lecture
	case based discussion
	practical
	test
Patient communication	lecture
	group discussion
	practical
Patient follow up	lecture
	group discussion
	practical
Casting	lecture
	video
	practical
Motivation for change	lecture
	case based discussion
	practical
Initial investigations	lecture
	case based discussion
	booklet

### Question 4. What practical skill should be assessed?

Participants ranked options in order of highest to lowest preference. Each option was given a score from 1–6 depending on how it was ranked by each participant. Scores for each option were tallied to determine the final rank order from highest to lowest preference amongst the group: 1. Initial assessment, 2. Debridement, 3. External fixation, 4. Patient communication, 5. Referral, 6. Patient follow up.

### Final course programme

The final two day course programme generated from the consensus meeting to run at the Malawi Orthopaedic Association Conference for 100 clinical officers is shown in Table 2.

**Table 2:** Final course programme

Day One	Day Two
08:30–09:00 Introduction and Pre-course Test	08:30–09.30 Post-operative Wound Care; Patient Communication and Follow up (lecture)
09:00–09:30 Motivational Speaker (lecture)	09.30–10:00 Casting (lecture)
09:30–10:00 Intro to Guidelines (lecture)	10:00–10:30 Break
10:30–11:00 Break	10.30–12:30 External Fixation (lecture and practical)
11:00–12:30 Assessment, Investigations (lecture)	12:30–13:30 Lunch
12:30–13:30 Lunch	13:30–14:30 Documentation and Referrals (lecture)
13.30–14:00 Antibiotics and Microbiology (lecture)	14:30–16:00 Written and Practical Tests
14:00–14:30 Anaesthesia (lecture)	16:00–16:30 Break
14:30–15:00 Break	16.30–17.00 Summary and Closing
15.00–17.00 Debridement, Grading, Closure (video)	

## Discussion

Existing established courses, such as Advanced Trauma Life Support and AO Fracture Management, as well as the WHO guidelines on surgical care at the district hospital include very little on open fractures [13–15]. This consensus meeting has resulted in a final course programme on open fracture management for clinical officers in Malawi, drawing on results from an audit on new guidelines and the expertise of orthopaedic surgeon educators. A number of strategies were employed to: 1. ensure the course was aligned to the MOA/AO Alliance guidelines while taking into account the trainee agenda, i.e. what they would find useful to learn, 2. encourage participant engagement despite possible perceived hierarchy and 3. reach a high level of agreement on decisions [16–18]. While initial presentations on the MOA/AO guidelines and results of the audit pushed through the trainer agenda, related course topics were suggested by participants indicating an agreement with the conclusions from these presentations. The suggestions also included some innovative ideas unrelated to the presentations, such as the topic “motivation for change” which would encourage clinical officers to engage with the course and improve their current practice.

Participants included surgeons and both senior and non-senior clinical officers from different districts in Malawi as well as surgeons from the UK. Due to this mix, it would be important to minimise the effect of any perceived hierarchy to create a more safe environment and maximise engagement from all meeting attendees [17, 18]. Ways in which this was achieved included giving participants the opportunity to get to know each other in an informal manner through a pre-meeting breakfast as well as other meals over the two days. The meeting itself started with attendee introductions and the room layout (a u-shape) allowed all members to see each other as well as the front of the room clearly so that no one would be excluded or forgotten at the back of the room.

The nominal group technique calls for methods to include everyone’s opinions. After each question was posed, participants were asked to begin making suggestions. As would be expected, some were more vocal than others and so sessions would include a “round-robin” (asking each participant to speak in turn in the order in which they are sitting) to ensure each had made a suggestion before discussing advantages and disadvantages. Course conveners, including both a surgeon and clinical officer, would be chairing sessions to conduct this. The anonymous nature of voting also ensured a high level of engagement that was evident in the response rate. To counter the fact that an option ranked low by a participant could become the highest ranking option overall, participants were given the chance to express disagreement with the outcome of the vote after each session. After the discussion, the vote could be repeated. This, however, did not have to be done during the course of the meeting.

Though these strategies helped strengthen the outcomes of this meeting, there are a few potential weaknesses to consider. The nominal group technique itself is less commonly used than the Delphi process in medical education [19] with disadvantages including limited rounds of voting, but, this is outweighed by allowing face to face discussions and anonymous voting with higher response rates than would have been achieved with the use of mailed questionnaires [10, 11]. The participants also only included surgeons and clinical officers yet nurses and plaster technicians are also involved in treating open fractures so there is a case for including these professionals in the consensus meeting as well as the resulting course.

It was anticipated that there may be a number of suggestions for pre-course and course material delivery methods, especially considering that conveners also presented a range of new and existing options. These included the use of digital mobile applications, online e-learning modules and practical simulation and moulage using prosthetic materials (e.g. open fracture limb sleeves). Discussion on these suggestions indicated that such methods would be costly and difficult to access in a setting where trainees do not always have the internet. This is consistent with retrospective reports in the literature from surgical skills courses set up in low and middle income countries, especially when this has been done in collaboration with high income countries [20–23]. The advantage to using a preliminary consensus meeting, or even a needs based assessment widely used in other work, is being able to anticipate and avoid such obstacles beforehand [24, 25].

As is clear from Table 1, participants preferred lectures as the delivery method for most topics, surprisingly even for the practical skill of casting for which participants were more interested in covering the theory. This raised concerns that a lecture heavy course may cause participants to lose interest during it and will fail to cater for different learning styles or allowing movement across learning stages. i.e. from watching to doing [26]. Though some courses heavily feature didactic teaching [20, 25], most other courses have aimed to keep a mix of teaching modalities [22–24]. When the possibility of diversifying the course was discussed with the group, they emphasised that if second or third ranked delivery options were selected for the final programme now or in later versions of the programme, such as practicals, they should be preceded by a lecture on the background and the material used should be sourced locally, i.e. the use of lamb legs would be preferable to expensive prostheses. This is also when the decision for the “external fixation” topic to have a lecture followed by a practical was made.

Conveners had already made the decision to evaluate the course through a number of methods, covering each stage of the Kirkpatrick model [27] (each stage is followed by an example): 1. reaction- participant feedback forms, 2) learning- testing: a) knowledge with written tests and b) practical skills with Objective Structured Assessment of Technical skills (OSAT) forms, 3) behaviour- monitoring application of learning i.e. how many steps of the open fracture management guidelines were followed before and after the course, 4) results- measuring impact of training on patient outcomes through an audit. The decision required of the meeting was for which practical skill should be tested specifically as logistically there would only be time for one. Contrary to convener expectation, the skill chosen was one that would be taught via a lecture rather than a practical session, “initial assessment”. This is an important aspect to cover but it may be necessary to override the consensus meeting on the delivery method for this topic so the course is better aligned and attendees are better prepared for the test [12]. 

## Conclusion

Overall, it is hoped that the outcome of this nominal group consensus meeting, and the resulting training, will improve access to treatment for open fractures in a way that is relevant and sustainable. Future work may build upon the findings to further enhance the course, include other health care professionals or adapt it for use in other countries.

## Data Availability

All data generated or analysed during this study are included in this published article.
